# Contribution of lower physical activity levels to higher risk of insulin resistance and associated metabolic disturbances in South Asians compared to Europeans

**DOI:** 10.1371/journal.pone.0216354

**Published:** 2019-05-07

**Authors:** Saima Afaq, Angad S. Kooner, Marie Loh, Jaspal S. Kooner, John C. Chambers

**Affiliations:** 1 Epidemiology and Biostatistics, Imperial College London, Norfolk Place, London, United Kingdom; 2 Institute of Public health and Social Sciences, Khyber medical university, Peshawar, Pakistan; 3 Hillingdon hospital, NHS Trust, Hillingdon, Greater London, United Kingdom; 4 Hammersmith Hospital, London, United Kingdom; 5 Ealing Hospital, Southall, Middlesex, United Kingdom; 6 NHLI, Imperial College London, Hammersmith Hospital, London, United Kingdom; 7 MRC-HPA Centre for Environment and Health, Imperial College London, Norfolk Place, London, United Kingdom; 8 Lee Kong Chian School of Medicine, Nanyang Technological University, Singapore, Singapore; McMaster University, CANADA

## Abstract

**Background:**

Insulin resistance and related metabolic disturbances are major risk factors for the higher T2D risk and associated morbidity and mortality amongst South Asians. The contribution of physical activity to the increased prevalence of insulin resistance and related disturbances amongst South Asians is unknown.

**Methods:**

We recruited 902 South Asian and European men and women, aged 35–85 years from the ongoing LOLIPOP study. Clinical characterisation comprised standardised questionnaire and measurement of height, weight, waist and hip circumference and blood pressure. Fasting bloods were taken for assessment of glucose, insulin, lipids and HbA1c. Physical activity was quantified using a validated accelerometer, Actigraph GT3X+, worn for 7 days. Univariate and multivariate approaches were used to investigate the relationship between ethnicity, physical activity, insulin resistance and related metabolic disturbances.

**Results:**

Total physical activity was ~31% (P = 0.01) lower amongst South Asians compared to Europeans (Mean MET.minutes [SD]: 1505.2 [52] vs. 2050.9 [86.6], P<0.001). After adjusting for age and sex, total physical activity had a negative association with HOMA-IR (B [SE]: -0.18 [0.08], P = 0.04) and fasting glucose levels (B[SE]: -0.11 [0.04], P = 0.02). There was no association between physical activity and other glycemic and lipid parameters. Total physical activity per week contributed towards the differences in insulin resistance and associated metabolic disturbances between South Asians and Europeans.

**Conclusion:**

Lower levels of physical activity may contribute to the increased insulin resistance in South Asians compared to Europeans. Our results suggest that lifestyle modification through increased physical activity may help to improve glucose metabolism and reduce the burden of excess T2D and related complications amongst South Asians.

## Background

People of South Asian ancestry (originating from India, Pakistan, Sri lanka and Bangladesh) are at high risk of type 2 diabetes (T2D). According to the IDF report 2015, worldwide there were an estimated 415 million South Asians with T2D in 2015, and this number is expected to increase to 642 million by 2040. Population studies from the non-migrant South Asians show that T2D affects ~15% of urban South Asians, compared to ~5% of their rural counterparts[1]. In the UK, prevalence of T2D amongst South Asians is reported to be up to three times higher than in the native European population (22% vs. 7%)[2].

The increased risk of T2D amongst South Asians is closely associated with the presence of insulin resistance and related metabolic disturbances (raised fasting insulin and glucose, central adiposity, high triglycerides and low high density lipid [HDL] cholesterol)[3]. Population studies report a higher prevalence of insulin resistance and related metabolic disturbances amongst South Asians compared to Europeans[2, 4]. This higher risk of insulin resistance and metabolic disturbances amongst UK South Asians is not explained by differences in common genetic variants[5], adiposity[6] or dietary macronutrient intake[7].

Observational and prospective studies show that increased physical activity is associated with improvement in insulin sensitivity and reduction in insulin resistance and related metabolic disturbances[8–12]. A reduction of ~7% (P<0.05) in fasting insulin levels has been reported for a 30-minute increase in moderate-intensity physical activity[13]. In addition, physical activity is reported to be associated with improvements in glucose metabolism, reduction in triglyceride concentration and an increase in high-density lipoprotein (HDL)[14, 15], even in the absence of weight loss[16, 17].

Cross-sectional studies in the UK report that South Asians have lower levels of physical activity than Europeans[18–20], raising the possibility that low levels of physical activity may contribute to the increased risk of T2D in South Asians. However, published studies have almost all used physical activity questionnaires, an approach known to be of limited accuracy and confounded by reporting bias[21]. Moreover, none of the questionnaires used for measuring physical activity levels have been validated for use amongst South Asians. Inaccurate quantification of physical activity levels amongst South Asians is a significant limitation to understanding the potential contribution of physical activity levels towards the higher risk of insulin resistance, associated metabolic disturbances and T2D in South Asians compared to Europeans.

Accelerometers are electronic sensors for objective measurement of physical activity, validated for use in free-living settings[22]. Compared to direct calorimetry and doubly-labelled water, accelerometers provide substantially greater accuracy for quantification of energy expenditure during physical activity than subjective methods (diaries, self-reports, questionnaires). In this study, we quantified physical activity by using accelerometer amongst South Asian and European men and women, to compare physical activity levels between the two populations, and to determine the contribution of physical activity to the higher prevalence of insulin resistance and related metabolic disturbances in South Asians.

## Methods

### Participants

We recruited 902 South Asian and European men and women, aged 35–85 years, from Dec 2013 till Oct 2015 to the study. All participants were recruited from the ongoing London Life Sciences Prospective Population (LOLIPOP) study amongst South Asians and Europeans. South Asians were defined as having all 4 grandparents born on the Indian subcontinent (India, Pakistan, Sri lanka and Bangladesh); Europeans were of self-reported white ancestry and born in the UK. People with musculoskeletal disorders which impaired habitual physical activity were excluded from the study. This study was approved by the London-Fulham Research Ethics Committee (ref 07/H0712/150), and all participants gave an informed written consent.

### Clinical characterisation

Participants attended a dedicated research clinic between 0900h and 1200h following an overnight fast. An interview-administered questionnaire was used to collect data on medical history, family history, current medications, personal and social history. Weight, height, waist circumference, hip circumference and blood pressure were measured by standardized protocols. Weight was measured to the nearest 0.1kg using digital scales mounted on a hard and flat surface. Height, waist and hip circumference were measured to the nearest 0.1cm. Height was measured using a stadiometer, mounted on a hard and flat surface. Waist circumference was defined as the point midway between the iliac crest and lowest rib. Hip circumference was defined as the maximum circumference over the greater trochanters and buttocks. Blood pressure was measured using an OMRON 705CP blood pressure monitor. Three measurements were taken and an average of these was documented.

Fasting venous blood samples were collected for glucose, insulin, lipid profile, and HbA1c. Blood samples were analysed in the Clinical Chemistry laboratories at Ealing Hospital NHS Trust, London, UK using the commercially available assays (**S1 Table**). Insulin resistance was assessed using the homeostatic model assessment (HOMA- IR), where HOMA-IR = [Glucose (mmol/L) x Insulin (mU/L)] / 22.5. HOMA-IR is a highly validated measure of insulin resistance which correlates closely with the gold standard euglycaemic-clamp method (r = 0.8, P<0.05)[23] for measurement of insulin resistance.

### Assessment of physical activity

Physical activity was measured objectively using Actigraph GT3X+, a tri-axial accelerometer that has been validated for assessment of free-living physical activity in Europeans, through comparison with gold standard doubly-labelled water and calorimetry. All participants were asked to wear the accelerometer for 7 consecutive days and nights (while sleeping), and only remove it during water based activities (swimming or bathing). The accelerometer was worn on the waist, using an elasticated belt. Participants were contacted on alternate days to ensure they were wearing the device. The accelerometers were returned at the end of the 7-day study period.

### Analysis of physical activity data

Accelerometer data were analysed using the manufacturer software (Actilife version 6.10.4). Non-wear time was identified and removed from analysis using the Troiano et al algorithm[24]. The algorithm identified periods of consecutive 60 minutes with zero acceleration recorded allowing a spike tolerance of 2 minutes, indicating that the device had not been worn during that hour. Participants who had worn the accelerometer for less than 7 days and for less than 10 hours per day (averaged over the 7 days) were excluded from final analysis.

Raw acceleration measured by the Actigraph GT3X+ was converted into counts, based on the vector magnitude (i.e. the sum of acceleration in all three axes). Counts are directly proportional to the frequency and intensity of the raw acceleration and hence the physical activity performed. Counts were used to classify data collected from the accelerometer into level of energy expenditure. The cut points defining different physical activity intensities were: (i) Light: 0–2690 counts per minute (CPM), (ii) Moderate: 2691–6166 CPM, (iii) Vigorous: 6167–9642 CPM, (iv) Very Vigorous: 9643 - ∞ CPM. Time (minutes per week) spent in different physical activity intensity categories was based on the above-mentioned cut points.

The Freedson VM3 Combination (2011) algorithm was used to calculate kilocalories consumed during physical activity over the 7-day period. This option combines the Freedson VM3 (2011) formula[25] with the Williams Work-Energy (1998) equation[26]. Freedson VM3 (2011) equation uses vector magnitude to estimate energy expenditure, which is only valid if the epoch counts exceed 2453 per minute. For counts less than 2453 per minute, Williams Work-Energy (1998) equation is used.

Metabolic Equivalents (METs) per minute were calculated from vertical axis counts using the Freedson adult (1998) equation[27]. Mets were then summed over the recording period to calculate MET.minutes in total physical activity over the 7-day period. MET.minutes were also calculated for light (METs<3), moderate (3–6 METS) and vigorous (METS>6) physical activity performed over the 7-day period.

### Statistical analysis

Statistical analyses were performed using SPSS version 22. Continuous data was expressed as mean ± standard deviation (SD), and categorical data as percentage (%). Distribution of the study variables was assessed using histograms. Univariate tests of the differences were carried out between South Asians and Europeans using the independent samples t-test and chi squared test for continuous and categorical variables respectively. Pearson’s correlation co-efficient was used for initial univariate assessment of the relationships between continuous variables. Finally, multivariate linear regression was used to investigate the relationships between ethnicity, physical activity, insulin resistance and related metabolic disturbances, taking account of potential confounding effects. All participants with type 2 diabetes were excluded from primary analysis.

## Results

### Characteristics of participants

We studied 902 South Asian and European men and women. Out of these, 219 participants did not wear accelerometer for at least 10 hours per day for 7 days and were excluded from analysis. This left 683 participants with complete data (183 Europeans and 500 South Asians), who were used for all analyses.

Clinical characteristics of the participants are shown in **Table 1**. South Asians were on average younger than Europeans (Mean [SD]: 53 [9] vs. 59 [10], P<0.001). Body mass index was similar (P = 0.3), but waist hip ratio higher in South Asians compared to Europeans (Mean [SD]: 0.95 [0.08] vs. 0.92 [0.08], P<0.001). South Asians had higher HOMA-IR, fasting glucose, HbA1c, and fasting insulin compared to Europeans (all P<0.001). Triglyceride levels were higher while HDL cholesterol was lower in South Asians than in Europeans (P<0.001). The prevalence of T2D was ~3 times higher in South Asians compared to Europeans (17.7% vs. 5.9%, P<0.001). Cigarette smoking and alcohol intake was lower amongst South Asians compared to Europeans (P<0.001).

**Table 1:** Clinical characteristics of South Asian and European participants. Results are presented as mean (SD) and percentages (%) for continuous and categorical variables respectively. P-values are determined from the independent student t-test (continuous variables) and Fisher’s exact test (categorical variables).

	Europeans	South Asians	
	Mean (SD)	Mean (SD)	p
N	183	500	
Age (years)	59.3 (10.1)	56.4 (9.0)	<0.001
Male (%)	42.7	53.1	0.02
Type 2 Diabetes (%)	5.9	17.7	<0.001
Hypertension (%)	25.9	35.7	0.01
Coronary heart disease (%)	6.5	8.3	0.4
Height (cm)	167.9 (8.8)	164.0 (9.4)	<0.001
Weight (kg)	78.7 (15.3)	74.1 (14.5)	<0.001
Body mass index (kg/m^2^)	27.8 (4.9)	27.4 (4.6)	0.3
Waist circumference (cm)	96.3 (12.6)	96.9 (11.7)	0.5
Hip circumference (cm)	104.3 (9.2)	101.9 (8.4)	0.001
Waist-hip ratio	0.92 (0.08)	0.95 (0.08)	<0.001
Systolic blood pressure (mmHg)	131.3 (17.6)	132.6 (17.4)	0.4
Diastolic blood pressure (mmHg)	78.0 (9.9)	79.2 (10.1)	0.2
Cholesterol (mmol/l)	5.20 (0.97)	4.95 (1.01)	0.002
Triglycerides (mmol/l)	1.18 (0.55)	1.46 (0.86)	<0.001
HDL cholesterol (mmol/l)	1.69 (0.45)	1.44 (0.42)	<0.001
LDL cholesterol (mmol/l)	2.98 (0.91)	2.85 (0.90)	0.07
Glucose (mmol/l)	5.28 (1.08)	5.87 (1.87)	<0.001
HbA1c (%)	5.70 (0.66)	6.21 (1.16)	<0.001
Insulin (mmol/l)	9.79 (6.16)	13.59 (9.39)	<0.001
HOMA-IR (mmol/l)	2.38 (1.71)	3.71 (3.10)	<0.001

### Physical activity levels in South Asians and Europeans

**Fig 1:**
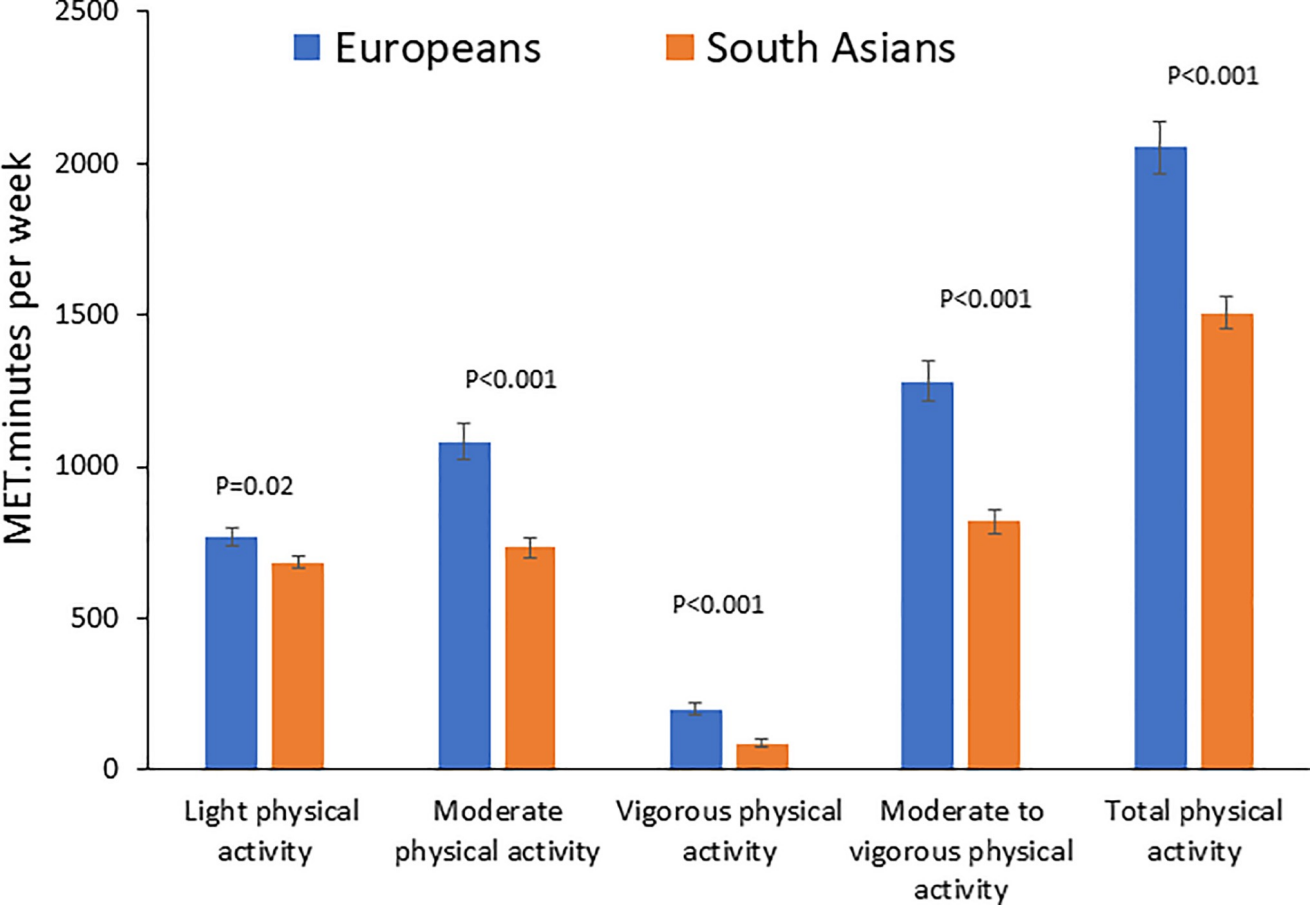
Physical activity levels (MET.minutes per week) amongst South Asians and Europeans. Error bars represent standard error of the mean. P-values are for the difference in physical activity between South Asians and Europeans in regression analysis with adjustment for age and sex.

Physical activity levels amongst South Asians and Europeans are summarised in **Fig 1.** Total physical activity levels expressed as MET.minutes per week were ~31% lower amongst South Asians than in Europeans (Mean MET.minutes [SD]: 1505.2 [52] vs. 2050.9 [86.6], P<0.001). In addition, compared to Europeans, South Asians showed lower levels of physical activity in all pre-specified intensity domains (light, moderate, vigorous and moderate to vigorous physical activity). Differences in age and sex did not contribute to the observed differences in physical activity between the two populations.

### Relationship of physical activity with insulin resistance

The univariate relationships between physical activity and insulin resistance, measured by HOMA-IR, showed a negative correlation between total physical activity levels per week and HOMA-IR (r = -0.1, P = 0.02, **S2 Table**). We used multiple regression analysis to evaluate the relationship between MET.minutes in total physical activity per week and HOMA-IR, after adjusting for differences in age, sex and ethnicity. Results revealed a negative association between total physical activity and HOMA-IR (B [SE]: -0.18 [0.08], P = 0.04, **Table 2**). Adjusting for adiposity (waist hip ratio) did not alter the association between physical activity and HOMA-IR (B [SE]: -0.20 [0.07], P = 0.01, **S3 Table**)

**Table 2:** Relationship of total physical activity with glucose, HbA1c, Insulin and HOMA-IR in regression analysis with adjustment for age, sex and ethnic group. Results are presented as the change (effect [SE]) in metabolic parameter per 1000 MET.minutes of total physical activity per week.

	Total MET.minutes per week Effect (SE)	p
Glucose (mmol/l)	-0.11 (0.04)	0.02
HbA1c (%)	-0.05 (0.03)	0.1
Insulin (IU/l)	-0.42 (0.28)	0.1
HOMA-IR (mmol/l)	-0.18 (0.08)	0.04

### Relationship of physical activity with glycaemic and lipid parameters

We investigated the relationship of MET.minutes in physical activity with biochemical measures of glucose and lipid metabolism. MET.minutes per week in total physical activity were inversely correlated with fasting glucose levels (r = -0.1, P<0.05) and HbA1c (r = -0.1, P<0.05). On the other hand, there was no significant correlation found between total physical activity and fasting insulin levels, HDL cholesterol or triglycerides (**S2 Table**). After adjusting for differences in age, sex and ethnicity, increased total physical activity per week was associated with lower fasting glucose levels (B[SE]: -0.11 [0.04], P = 0.02, **Table 2**). The association between fasting glucose levels and physical activity per week remained significant after additional adjustment for waist hip ratio (B[SE]: -0.11 [0.04], P = 0.01, **S3 Table**). Multiple regression analysis showed no evidence for a relationship of total physical activity with HbA1c (B[SE]: -0.05 (0.03), P = 0.1) or fasting insulin levels (B[SE]: -0.42 (0.28), P = 0.1, **Table 2**).

### Sensitivity analysis

We carried out sensitivity analyses to evaluate the robustness of our findings. Analysis of physical activity levels amongst South Asians and Europeans showed similar results using alternate metrics of physical activity as a sensitivity analysis (total counts, kilocalories and time spent in total physical activity, **S4 Table**). The relationships of physical activity with HOMA-IR, glycaemic parameters and lipid parameters were not materially altered using alternate classification schemes for physical activity, log-transformation of positively skewed variables or by inclusion of people with T2D (**S5, S6 and S7 Tables**).

### Contribution of physical activity to metabolic disturbances in South Asians compared to Europeans

We used multiple regression to quantify the contribution of physical activity to metabolic disturbances amongst South Asians compared to Europeans (**Table 3**). Results are expressed as difference in respective metabolic parameters amongst South Asians and Europeans, after adjustment for potential predictors. Results showed that glucose concentrations were 0.17mmol/L (SE:0.08, P = 0.03) higher amongst South Asians than Europeans. After adjusting for total physical activity, the difference in fasting glucose between South Asians and Europeans was reduced to up to 0.13mmol/L (SE:0.08) and was no longer statistically significant. In contrast, physical activity, although associated with a decrease in HOMA-IR, did not account for the differences in HOMA-IR and other glycaemic and lipid parameters between South Asians and Europeans (**Table 3**).

**Table 3:** Contribution of physical activity towards the excess levels of metabolic disturbances amongst South Asians compared to Europeans. Beta coefficient from multiple linear regression represents the difference in metabolic parameters between South Asians (SA) and Europeans (EW) before and after adjustment for total physical activity (MET.minutes) per week.

Metabolic parameters	Unadjusted for physical activity	p	Adjusted for total MET.minutes/week	p
Glucose	0.17 (0.08)	0.03	0.13 (0.08)	0.1
HbA1c	0.29 (0.05)	<0.001	0.27 (0.05)	<0.001
Insulin	3.2 (0.8)	<0.001	2.9 (0.8)	<0.001
HOMA-IR	0.9 (0.2)	<0.001	0.8 (0.2)	<0.001
Cholesterol	-0.2 (0.09)	0.03	-0.2 (0.09)	0.04
Triglycerides	0.2 (0.07)	0.002	0.2 (0.07)	0.006
HDL	-0.2 (0.04)	<0.001	-0.2 (0.04)	<0.001
LDL	-0.1 (0.08)	0.1	-0.1 (0.08)	0.2

## Discussion

Our results show that total physical activity levels are ~31% lower amongst South Asians than Europeans in the UK. Total physical activity is independently and inversely associated with glucose and insulin resistance but not with other metabolic parameters. Low levels of physical activity do not account for the higher levels of insulin resistance amongst South Asians. However, lower physical activity levels amongst South Asians may contribute to their elevated glucose concentrations compared to Europeans. Our results suggest that lifestyle modification through increased physical activity may help to improve glucose metabolism and thus reduce the excess burden of T2D amongst South Asians.

The ~3 fold higher prevalence of type 2 diabetes in South Asians compared to Europeans in our study, is consistent with the existing literature. The Southall and Brent Revisited Study (SABRE) study, 20 year follow-up study of participants in the Southall study, reported that the prevalence of T2D was 22% amongst South Asians compared to 7% amongst Europeans[2]. Holman et al[28] compared the prevalence of T2D between South Asians and Europeans living in England in 2010. Results from this study estimated that the prevalence of T2D was ~2 times higher in South Asians compared to Europeans (14.0% vs. 6.9% respectively). A meta-analysis of international epidemiological studies and health examination surveys, by Danaei et al[29], showed similar results. Our findings thus reaffirm the high burden of type 2 diabetes in the South Asian population.

Though previous studies have suggested that levels of leisure time and total physical activity are lower amongst South Asians than Europeans, they have exclusively used semi-quantitative methods that are not validated amongst South Asians[18, 30, 31]. A recent study reported low levels of physical activity amongst South Asians compared to Europeans but was carried out in a population sample with low response rate (<5%) and exclusively male, and therefore unrepresentative of the UK South Asian population[32]. In the present study, we used a validated tri- axial accelerometer[33] to quantify physical activity levels amongst a representative population sample of South Asian and European men and women recruited from general practitioner lists (response rates averaged 62% in South Asians and 61% in Europeans). We show that total physical activity levels are ~31% lower amongst South Asians than Europeans, as measured by energy expenditure during free living activity. Lower levels of physical activity amongst South Asians were most evident in the moderate and vigorous physical activity domains, but also present in light physical activity. Low levels of physical activity amongst South Asians were independent of and not explained by differences in age, sex or adiposity, compared to Europeans. Our results extend current knowledge by providing quantitative assessment of physical activity in a representative sample of South Asian men and women. The reasons underlying low levels of physical activity amongst South Asians are not known, but may include a lack of understanding regarding the health benefits associated with regular physical activity, as well as cultural expectations and barriers to participation in recreational activities, especially among South Asian women[34].

We did not find an association of physical activity with HbA1c, triglycerides or HDL cholesterol concentrations. However, our results do show an association of lower levels of physical activity with higher insulin resistance and fasting glucose concentrations. In regression analysis, the relationship of physical activity with insulin resistance and glucose was not accounted for by differences in age, sex or adiposity, suggesting that physical activity has an independent effect on insulin resistance and fasting glucose concentrations. Our results are consistent with published data which show that increased physical activity is associated with a reduction in insulin resistance and glucose concentrations in European populations[35–38]. Findings of the current study are in line with experimental studies which show that physical activity stimulates translocation of GLUT4 glucose transporters, the rate-limiting step in glucose metabolism, in skeletal muscle[39].

Our results show that insulin resistance, as measured by HOMA-IR, and related insulin and lipid disturbances are more common amongst South Asians than Europeans. Lower physical activity alone did not explain the presence of these metabolic abnormalities amongst South Asians. However, we found that insulin resistance and glucose concentrations were reduced significantly after adjusting for total physical activity. Our findings suggest that higher concentrations of glucose amongst South Asians are in part accounted for by their lower levels of physical activity compared to Europeans. Abnormal glucose metabolism underlies T2D and impaired glucose tolerance, both major risk factors for cardiovascular disease. Prospective studies show that elevated glucose despite being in the normal range is a risk factor for cardiovascular disease[2, 40]. Low physical activity amongst South Asians may therefore have a role in the increased risk of cardiovascular disease in this population, through an effect on glucose metabolism.

This is the first study to quantify physical activity levels using an objective, validated tool in a representative sample of South Asian and European men and women. However, this study does have some limitations. Our study is cross-sectional rather than prospective and so may be susceptible to confounding effects (“reverse causation”). It is possible that our sample of participants may not be fully representative of the spectrum of South Asian and European populations. This is unlikely though as participants were recruited from general practitioner lists and showed the well-established patterns of insulin resistance and related metabolic abnormalities amongst South Asians. Modest sample size is a potential explanation for the failure to find the previously reported associations of physical activity with fasting insulin levels and related lipid abnormalities though this relationship is not consistently identified in other studies[41, 42]. We also used HOMA-IR, a surrogate measure of insulin resistance, which is closely correlated with the gold standard Hyperinsulinaemic euglycaemic clamp but which remains a less accurate measure of insulin resistance. Although physical activity has been measured objectively in the present study, ‘cardio respiratory fitness’, which has been indicated as a strong predictor of metabolic health, was not determined[43]. Furthermore, the detrimental effect of smoking on cardiorespiratory fitness and physical activity is well established[44]. Future research should consider including this important risk factor in the analysis too. Finally, although accelerometers are an objective measure of physical activity and their use has been validated against the gold standard under close supervision, their accuracy in free living individuals is lower, and our estimates of physical activity may be imprecise. In addition, the “regression dilution”[45] bias might have underestimated the strength of relationship between physical activity and metabolic parameters. To avoid the regression dilution bias, prospective studies should be conducted where physical activity levels are measured on more than one occasion.

In conclusion, we show that physical activity levels are lower amongst South Asians and may contribute to their increased fasting glucose concentrations and insulin resistance, compared with Europeans. Our results suggest the potential for increased physical activity to improve glucose metabolism and insulin resistance amongst South Asians, and help reduce their excess risk of T2D and cardiovascular disease.

## Supporting information

S1 Table Methods used for biochemical analysis. (DOCX)

S2 Table Correlation (Pearson’s correlation) of total physical activity energy expenditure (MET.minutes) with insulin resistance and related metabolic disturbances. (DOCX)

S3 Table Effect of adiposity (waist hip ratio) on the relationship between physical activity MET.minutes and fasting glucose levels and HOMA-IR. Results are presented as the change (effect [SE]) in glucose levels for a 1000 MET.minutes per week increase in physical activity after adjustment for age, sex, ethnicity and waist hip ratio.
(DOCX)

S4 Table Physical activity levels amongst South Asians and Europeans. Similar results were obtained using alternate metrics of physical activity as a sensitivity analysis (total counts, kilocalories and time total physical activity per week).
(DOCX)

S5 Table Relationship of alternate measures of physical activity with glucose, HbA1c, insulin and HOMA-IR in regression analysis with adjustment for age, sex and ethnic group. (DOCX)

S6 Table Relationship of physical activity with glucose in regression analysis with adjustment for age, sex and ethnic group. Outcome variables, with a positively skewed distribution, have been log-transformed.
(DOCX)

S7 Table Relationship of physical activity with glucose and HbA1c in regression analysis with adjustment for age, sex and ethnic group. T2D cases have also been included in the analysis. Results are presented as the change (effect [SE]) in glucose or HbA1c for physical activity per week: *per 100000 counts/week, **per 100 minutes/week, ***per 1000 kilocalories or MET.minutes/week.
(DOCX)
