# Supplementary material for: Contribution of lower physical activity levels to higher risk of insulin resistance and associated metabolic disturbances in South Asians compared to Europeans
Source: PLoS One. 2019 May 7;14(5):e0216354. doi: 10.1371/journal.pone.0216354 (PMC6504088; doi:10.1371/journal.pone.0216354)
Supplement: S1 Table — (DOCX) [file pone.0216354.s001.docx]

Supporting Information

**Contribution of lower physical activity levels to higher risk of Insulin resistance and associated metabolic disturbances in South Asians compared to Europeans.**

**S1 Table.** Methods used for biochemical analysis.

|  | **Method** | **Analyser** |
| --- | --- | --- |
| **Glucose** | Enzymatic, colorimetric method | Roche/ Hitachi cobas c 501 |
| **Insulin** | Sandwich principle | Cobas e 411 |
| **Cholesterol** | Enzymatic reference method with hexokinase | Roche/ Hitachi cobas c 501 |
| **Triglycerides** | Enzymatic colorimetric test | Roche/ Hitachi cobas c 501 |
| **HDL-C** | Homogeneous enzymatic colorimetric test | Roche/ Hitachi cobas c 501 |
| **HbA1c** | High performance liquid chromatography | Bio-Rad VARIANTTM II TURBO |
