# Supplementary material for: Contribution of lower physical activity levels to higher risk of insulin resistance and associated metabolic disturbances in South Asians compared to Europeans
Source: PLoS One. 2019 May 7;14(5):e0216354. doi: 10.1371/journal.pone.0216354 (PMC6504088; doi:10.1371/journal.pone.0216354)
Supplement: S2 Table — (DOCX) [file pone.0216354.s002.docx]

Supporting Information

**Contribution of lower physical activity levels to higher risk of Insulin resistance and associated metabolic disturbances in South Asians compared to Europeans.**

**S2 Table.** Correlation (Pearson’s correlation) of total physical activity energy expenditure (MET.minutes) with insulin resistance and related metabolic disturbances.

|  | **Total physical activity**  **r** | **p** |
| --- | --- | --- |
| Glucose | -0.11 | 0.006 |
|  |  |  |
| HbA1c | -0.13 | 0.001 |
|  |  |  |
| Triglycerides | -0.07 | 0.07 |
|  |  |  |
| HDL | 0.04 | 0.3 |
|  |  |  |
| Insulin | -0.06 | 0.1 |
|  |  |  |
| HOMA-IR | -0.09 | 0.02 |
|  |  |  |
