# Supplementary material for: Contribution of lower physical activity levels to higher risk of insulin resistance and associated metabolic disturbances in South Asians compared to Europeans
Source: PLoS One. 2019 May 7;14(5):e0216354. doi: 10.1371/journal.pone.0216354 (PMC6504088; doi:10.1371/journal.pone.0216354)
Supplement: S3 Table — Results are presented as the change (effect [SE]) in glucose levels for a 1000 MET.minutes per week increase in physical activity after adjustment for age, sex, ethnicity and waist hip ratio. (DOCX) [file pone.0216354.s003.docx]

Supporting Information

**Contribution of lower physical activity levels to higher risk of Insulin resistance and associated metabolic disturbances in South Asians compared to Europeans.**

**S3 Table.** Effect of adiposity (waist hip ratio) on the relationship between physical activity MET.minutes and fasting glucose levels and HOMA-IR. Results are presented as the change (effect [SE]) in glucose levels for a 1000 MET.minutes per week increase in physical activity after adjustment for age, sex, ethnicity and waist hip ratio.

|  | **Total MET.minutes per week** | **p** |
| --- | --- | --- |
| Glucose (mmol/l) | -0.11 (0.04) | 0.01 |
|  |  |  |
| HOMA-IR (mmol/l) | -0.20 (0.07) | 0.01 |
