# Supplementary material for: Contribution of lower physical activity levels to higher risk of insulin resistance and associated metabolic disturbances in South Asians compared to Europeans
Source: PLoS One. 2019 May 7;14(5):e0216354. doi: 10.1371/journal.pone.0216354 (PMC6504088; doi:10.1371/journal.pone.0216354)
Supplement: S4 Table — Similar results were obtained using alternate metrics of physical activity as a sensitivity analysis (total counts, kilocalories and time total physical activity per week). (DOCX) [file pone.0216354.s004.docx]

Supporting Information

**Contribution of lower physical activity levels to higher risk of Insulin resistance and associated metabolic disturbances in South Asians compared to Europeans.**

**S4 Table.** Physical activity levels amongst South Asians and Europeans. Similar results were obtained using alternate metrics of physical activity as a sensitivity analysis (total counts, kilocalories and time total physical activity per week).

|  | **Europeans** | | **South Asians** | | **p** |
| --- | --- | --- | --- | --- | --- |
|  | **Mean** (**SD)** | | **Mean** **(SD)** | |  |
|  |  |  |  |  |  |
| *Total counts per week* |  |  |  |  |  |
| Vector magnitude counts | 4157254.9 (125344.9) | | 3793311.0 (75306.4) | | 0.01 |
| Vertical axis counts | 2280907.8 (72952.9) | | 1946203.9 (43829.7) | | <0.001 |
| Minutes in total physical activity | 7625.2 (78.9) | | 7891.6 (47.4) | | 0.004 |
| Kilocalories per week | 4156.6 (152.9) | | 3248.9 (91.8) | | <0.001 |
|  |  | |  | |  |
