# Supplementary material for: Contribution of lower physical activity levels to higher risk of insulin resistance and associated metabolic disturbances in South Asians compared to Europeans
Source: PLoS One. 2019 May 7;14(5):e0216354. doi: 10.1371/journal.pone.0216354 (PMC6504088; doi:10.1371/journal.pone.0216354)
Supplement: S5 Table — (DOCX) [file pone.0216354.s005.docx]

Supporting Information

**Contribution of lower physical activity levels to higher risk of Insulin resistance and associated metabolic disturbances in South Asians compared to Europeans.**

**S5 Table.** Relationship of alternate measures of physical activity with glucose, HbA1c, insulin and HOMA-IR in regression analysis with adjustment for age, sex and ethnic group.

|  | **Glucose (mmol/l)** | |  | **HbA1c (%)** | |  | **Insulin (IU/l)** | |  | **HOMA-IR (mmol/l)** | |
| --- | --- | --- | --- | --- | --- | --- | --- | --- | --- | --- | --- |
|  | **Effect (SE)** | **p** |  | **Effect (SE)** | **p** |  | **Effect (SE)** | **p** |  | **Effect (SE)** | **p** |
| Total counts per week | | | | | |  |  |  |  |  |  |
| Vector Magnitude Counts* | -0.01 (0.003) | 0.002 |  | -0.01 (0.002) | 0.02 |  | -0.07 (0.01) | 0.001 |  | -0.02 (0.001) | <0.001 |
| Vertical axis counts* | -0.02 (0.005) | 0.003 |  | -0.01(0.003) | 0.03 |  | -0.10 (0.04) | 0.003 |  | -0.03 (0.01) | 0.001 |
| Minutes in total physical activity** | -0.14 (0.05) | 0.01 |  | -0.04 (0.03) | 0.1 |  | -0.09 (0.03) | 0.8 |  | -0.1 (0.04) | 0.02 |
| Kilocalories per week*** | -0.05 (0.03) | 0.05 |  | -0.02 (0.02) | 0.3 |  | -0.02 (0.02) | 0.3 |  | 0.01 (0.05) | 0.8 |

*Results are presented as the change (effect [SE]) in metabolic parameter for physical activity levels per week:*

**per 100000 counts/week*

***per 100minutes/week*

****per 1000 kilocalories/week*
