# Supplementary material for: Contribution of lower physical activity levels to higher risk of insulin resistance and associated metabolic disturbances in South Asians compared to Europeans
Source: PLoS One. 2019 May 7;14(5):e0216354. doi: 10.1371/journal.pone.0216354 (PMC6504088; doi:10.1371/journal.pone.0216354)
Supplement: S6 Table — Outcome variables, with a positively skewed distribution, have been log-transformed. (DOCX) [file pone.0216354.s006.docx]

Supporting Information

**Contribution of lower physical activity levels to higher risk of Insulin resistance and associated metabolic disturbances in South Asians compared to Europeans.**

**S6 Table.** Relationship of physical activity with glucose in regression analysis with adjustment for age, sex and ethnic group. Outcome variables, with a positively skewed distribution, have been log-transformed.

|  |  | **% decrease in Glucose levels (95% CI)** | **p** |
| --- | --- | --- | --- |
| **Total counts per week** |  |  |  |
| Vector Magnitude Counts |  | 0.13 (0.05-0.21) | 0.001 |
| Vertical axis counts |  | 0.20 (0.07-0.32) | 0.002 |
| Minutes in total physical activity |  | 0.13 (0.01-0.25) | 0.03 |
| Kilocalories per week |  | 0.54 (0.08-1.16) | 0.09 |
| Total MET.minutes in physical activity |  | 1.39 (0.30-2.47) | 0.01 |

*Results are presented as the percentage decrease in glucose levels for physical activity levels per week:*

**per 100000 counts/week*

***per 100minutes/week*

****per 1000 kilocalories or Met.minutes/week*
