# Supplementary material for: Contribution of lower physical activity levels to higher risk of insulin resistance and associated metabolic disturbances in South Asians compared to Europeans
Source: PLoS One. 2019 May 7;14(5):e0216354. doi: 10.1371/journal.pone.0216354 (PMC6504088; doi:10.1371/journal.pone.0216354)
Supplement: S7 Table — T2D cases have also been included in the analysis. Results are presented as the change (effect [SE]) in glucose or HbA1c for physical activity per week: *per 100000 counts/week, **per 100 minutes/week, ***per 1000 kilocalories or MET.minutes/week. (DOCX) [file pone.0216354.s007.docx]

Supporting Information

**Contribution of lower physical activity levels to higher risk of Insulin resistance and associated metabolic disturbances in South Asians compared to Europeans.**

**S7 Table.** Relationship of physical activity with glucose and HbA1c in regression analysis with adjustment for age, sex and ethnic group. T2D cases have also been included in the analysis. Results are presented as the change (effect [SE]) in glucose or HbA1c for physical activity per week: *per 100000 counts/week, **per 100 minutes/week, ***per 1000 kilocalories or MET.minutes/week.

|  | **Glucose (mmol/l)** | |  | **HbA1c (%)** | |  | **Insulin (IU/l)** | |  | **HOMA-IR (mmol/l)** | |
| --- | --- | --- | --- | --- | --- | --- | --- | --- | --- | --- | --- |
|  | **Effect (SE)** | **P** |  | **Effect (SE)** | **P** |  | **Effect (SE)** | **P** |  | **Effect (SE)** | **P** |
| **Total counts per week** | | | | | |  |  |  |  |  |  |
| Vector Magnitude Counts* | -0.01 (0.004) | 0.005 |  | -0.01 (0.002) | 0.02 |  | -0.06 (0.01) | <0.001 |  | -0.03 (0.001) | <0.001 |
| Vertical axis counts* | -0.02 (0.007) | 0.02 |  | -0.01 (0.003) | 0.03 |  | -0.09 (0.03) | 0.004 |  | -0.04 (0.01) | 0.001 |
| **Minutes in physical activity per week**** | | | | | |  |  |  |  |  |  |
| Light physical activity | -0.13 (0.06) | 0.05 |  | -0.05 (0.03) | 0.2 |  | -0.03 (0.03) | 0.3 |  | -0.004 (0.01) | 0.7 |
| Moderate physical activity | -0.49 (0.29) | 0.02 |  | -0.2 (0.14) | 0.1 |  | -0.3 (0.01) | 0.04 |  | -0.1 (0.04) | 0.02 |
| Vigorous physical activity | -1.39 (0.91) | 0.1 |  | -0.68 (0.4) | 0.5 |  | -0.4 (0.5) | 0.4 |  | -0.2 (0.2) | 0.3 |
| Moderate to vigorous physical activity | -0.44 (0.24) | 0.02 |  | -0.18 (0.11) | 0.1 |  | -0.2 (0.1) | 0.05 |  | -0.1 (0.04) | 0.03 |
| Total physical activity | -0.15 (0.06) | 0.02 |  | -0.06 (0.03) | 0.1 |  | -0.03 (0.01) | 0.7 |  | -0.01 (0.01) | 0.4 |
| **Energy Expenditure per week***** | | | | | |  |  |  |  |  |  |
| Kilocalories per week | -0.05 (0.03) | 0.09 |  | -0.03 (0.02) | 0.3 |  | -0.2 (0.2) | 0.3 |  | 0.01 (0.01) | 0.8 |
| MET.minutes per week |  | | | | |  |  |  |  |  |  |
| Light physical activity | -0.1 (0.16) | 0.5 |  | -0.1 (0.07) | 0.3 |  | -2.0 | 0.02 |  | -1.0 | 0.03 |
| Moderate physical activity | -0.15 (0.08) | 0.03 |  | -0.06 (0.04) | 0.1 |  | -0.3 | 0.5 |  | -0.2 | 0.2 |
| Vigorous physical activity | -0.51 (0.24) | 0.04 |  | -0.26 (0.12) | 0.3 |  | -1.0 | 0.6 |  | -0.5 | 0.3 |
| Moderate to vigorous physical activity | -0.16 (0.07) | 0.03 |  | -0.06 (0.03) | 0.09 |  | -0.3 | 0.4 |  | -0.2 | 0.2 |
| Total physical activity | -0.11 (0.06) | 0.03 |  | -0.05 (0.03) | 0.08 |  | -0.4 | 0.1 |  | -0.2 | 0.05 |
